# Distal C Terminus of Ca_V_1.2 Channels Plays a Crucial Role in the Neural Differentiation of Dental Pulp Stem Cells

**DOI:** 10.1371/journal.pone.0081332

**Published:** 2013-11-21

**Authors:** Jianping Ge, Yanqin Ju, Zhigang Xue, Yun Feng, Xiaofeng Huang, Hongwei Liu, Shouliang Zhao

**Affiliations:** 1 Department of Conservative Dentistry, Laboratory of Oral Biomedical Science and Translational Medicine, School of Stomatology, Tongji University, Shanghai, China; 2 Department of Regenerative Medicine, Stem Cell Research Center, Translational Center for Stem Cell Research, Tongji Hospital, School of Medicine, Tongji University, Shanghai, China; Instituto Butantan, Brazil

## Abstract

L-type voltage-dependent Ca_V_1.2 channels play an important role in the maintenance of intracellular calcium homeostasis, and influence multiple cellular processes. C-terminal cleavage of Ca_V_1.2 channels was reported in several types of excitable cells, but its expression and possible roles in non-excitable cells is still not clear. The aim of this study was to determine whether distal C-terminal fragment of Ca_V_1.2 channels is present in rat dental pulp stem cells and its possible role in the neural differentiation of rat dental pulp stem cells. We generated stable Ca_V_1.2 knockdown cells via short hairpin RNA (shRNA). Rat dental pulp stem cells with deleted distal C-terminal of Ca_V_1.2 channels lost the potential of differentiation to neural cells. Re-expression of distal C-terminal of Ca_V_1.2 rescued the effect of knocking down the endogenous Ca_V_1.2 on the neural differentiation of rat dental pulp stem cells, indicating that the distal C-terminal of Ca_V_1.2 is required for neural differentiation of rat dental pulp stem cells. These results provide new insights into the role of voltage-gated Ca^2+^ channels in stem cells during differentiation.

## Introduction

Dental pulp stem cells (DPSCs) are a part of dental mesenchyme and are derived from cranial neural crest cells [1,2]. In vivo and in vitro studies have shown that dental stem cells have neural differentiation capacity under proper culture condition [3-6]. And it was recently reported that DPSCs demonstrate better neural and epithelial stem cell properties than bone marrow-derived mesenchymal stem cells [7-11]. These studies suggest the potential uses of dental stem cells in the field of neurodegenerative and oral diseases in the future. However, the molecular regulation of differentiation of dental pulp stem cells is not well understood. 

Changes in intracellular Ca^2+^ concentration ([Ca^2+^]i) play a central role in neuronal differentiation. Ca^2+^ influx into cells can generate biological signals, which can modulate expression of genes involving in cell proliferation and neuronal differentiation. Among the ten different types of voltage gated calcium channels, voltage-gated L-type Ca^2+^ channels (LTCs) are particularly effective at inducing changes in gene expressions [12-16]. Studies in neurons and cardiac myocytes have suggested that the C terminus of Ca_V_1.2 is proteolytically cleaved, yielding a truncated channel and a cytoplasmic C-terminal fragment, which is also called the distal C-terminus (DCT). In neurons DCT regulates transcription of a variety of genes, and interacts with nuclear proteins and stimulates neurite outgrowth [17-20]. DCT has also been reported to repress the expression of Ca_V_1.2, suggesting it as an important factor of auto-feedback regulatory pathway [21]. However the expression of DCT and its possible role in dental pulp stem cells is still unclear.

We hypothesized that DCT of Ca_V_1.2 channels plays a significant role in orienting DPSCs differentiation toward the neuronal phenotype. We thus investigated the neural differentiation of rDPSCs in vitro to determine whether DCT of Ca_V_1.2 channels regulates the differentiation properties of DPSCs. We generated stable Ca_V_1.2 knockdown cells via short hairpin RNA (shRNA). These cells were then used in neural differentiation experiments. Our results showed that in Ca_V_1.2 knock-down rDPSCs neural differentiation was significantly decreased. And re-expression of DCT rescued the effect of knocking down the endogenous Ca_V_1.2 on the neural differentiation of rDPSCs, indicating that the DCT of Ca_V_1.2 channels is required for neural differentiation of DPSCs.

## Methods

### Ethical approval

All animal studies were approved by the Institutional Animal Care and Use Committee of Tongji University, Shanghai, China.

### Isolation and culture of rDPSCs

rDPSCs of the incisors were harvested from the dental pulp of postnatal week 3 Sprague–Dawley rat (Harlan Sprague Dawley, Shanghai, China) and cultured according to published methods [3]. Special care was taken to prevent microbial contamination as well as contamination by other dental cell populations. Briefly, the rat’s mandible was removed and all soft tissue was blunt-dissected away to reveal the incisor insertion. The incisors were then extracted from the mandible. Any loose tissue on the root ends of the teeth was trimmed off and the external portions of the teeth were sterilized via immersion in 1% povidone–iodine for 2 min, followed by immersion in 0.1% sodium thiosulphate for 1 min and then a final rinse in sterile PBS. The pulp was removed from each tooth and placed in an enzymatic bath consisting of a mixture of 3mg/ml collagenase type I and 4mg/ml dispase (Sigma). After a 40 min incubation period at 37°C , the enzymes were neutralized with 10% serum in culture medium and the pulp digest was centrifuged at 500 × g for 5 min to yield a cell pellet, which was then re-suspended in fresh culture medium and passed through a 70μm strainer (Falcon, Gibco) to obtain single-cell suspension. Cells were seeded at a density of 10^4^cells/cm^2^ in culture dishes. Cells were grown in α-MEM supplemented with 100 mM ascorbic acid 2-phosphate, 2 mM GluMAX (Gibco), 100 U/ml penicillin, 100 mg/ml streptomycin, 10% fetal bovine serum(FBS, Gibco) and aerated with 5% CO_2_ at 37°C. Experiments were performed with cells from passages 3 through 5. 

### Antibody Generation

Rabbit polyclonal antibody against the distal C terminus (anti-DCT) was generated against residues 2106–2120 in the distal C terminus of Ca_V_1.2 channels and characterized as previously described[20]. Peptides of the following sequence DPGQDRAVVPEDES were synthesized, coupled to KLH, injected into rabbits, and affinity purified by GL Biochem.

### Plasmid Construction, Lentivirus packaging and transduction in rat DPSCs

The recombinant DCT sequences encompassing amino acids 1642-2143 of the Ca_V_1.2 (accession # AAA18905) were constructed by inserting PCR amplified portions of the Ca_V_1.2 C-terminal tail into the EcoI/BamHI sites of pLVX-IRES-ZsGreen1 vector (Clontech). 

Lentivirus shRNA was constructed into the BamHI/EcoI sites of pLVX-shRNA2 Vector (Clontech) as previously described [22,23]. Restriction sites were added to ends of the sense and anti-sense oligo’s which contained a loop and a termination signal. The oligo’s designed were synthesized from Invitrogen with a 50 phosphate and PAGE purified. The oligo format is the following .Sense Oligo: 5'-GATCCGCCATTTTCACCATTGAAATTTCAAGAGAATTTCAATGGTGAAAATGGTTTTTTG-----3'. Anti-sense oligo: 5'-AATTCAAAAAACCATTTTCACCATTGAAATTCTCTTGAAATTTCAATGGTGAAAATGGCG-----3'. The luciferase-shRNA is as negative control. The oligo format is the following. Sense Oligo: 5'-GATCCGATATTGCTGCGATTAGTCTTCAAGAGAGACTAATCGCAGCAATATCTTTTTTACGCGTG-3'. Anti-sense oligo: 5'-AATTCACGCGTAAAAAAGATATTGCTGCGATTAGTCTCTCTTGAAGACTAATCGCAGCAATATCG-3'. Lentivirus packaging, purification, and titer determination of the lentivirus were performed as described previously [23]. The DCT vector or sh vector or luc vector were co-cultured with the lentiviral packaging vectors pRSV-REV, pMDLg/RRE, and the vesicular stomatitis virus G glycoprotein (VSVG) by Ca2+ phosphate transfection of HEK293T cells using standard protocols to obtain the recombinant viruses. At day 3 post-culture, rDPSCs were incubated with different lentiviruses for 3-5 days. Cells transduction efficiency was determined by FACScan flow cytometry (BD Biosciences, Franklin Lakes, NJ) to sort GFP^+^ cells. Stably transduced cells were used for functional experiments.

### Induction and Detection of Cell Differentiation

Neural differentiation of rDPSCs was done as previously described [3]. Briefly, cells were reseeded at a density of 1×10^5^/well in 2 ml/well growth media in 6-well plates. For 3 days, cells were washed with phosphate-buffered saline(PBS) and then cultured in Neurobasal Media ( Invitrogen, Carlsbad, CA) for 2 weeks, which consists of 100U/ml penicillin, 100U/ml streptomycin, 1×B27 supplement, 20ng/ml epidermal growth factor (EGF,400-15; PeproTech), and 40 ng/ml basic fibroblast growth factor (FGF, 400-29; ProspecTech). Control samples were maintained in growth media for the duration of the neuronal inductive assay. The media for all conditions were replaced every 3 days. Following the final incubation, the 6-well coated chamber slides were fixed with 4% paraformaldehyde (PFA), and cells in 6-well plates were lysed with TRizol (Invitrogen) and stored for RNA isolation and real time-polymerase chain reaction (RT-PCR) analysis.

### Isolation of RNA and real-time RT-PCR

Total RNA was extracted using the TRIzol method. RNA samples were quantified by spectrophotometer (Eppendorf, Hamburg, Germany), and RNA integrity was checked on 1% agarose gels using a deionized formamide-based loading buffer. Reverse-transcription reactions were performed using Superscript III reverse transcriptase (Invitrogen). cDNA samples were diluted to a uniform concentration of 50 ng/μl. Real-time PCR was performed with an ABI 7500 real-time PCR system (Applied Biosystem) using the SYBR Premix Ex Taq^TM^ (Perfect Real Time) Sample (TaKaRa, Japan). After the real-time PCR procedure, a Ct value was obtained for each sample. This Ct value showed how many PCR cycles were necessary to reach a certain level of fluorescence. The amplification efficiency of different genes was determined relative to GAPDH as an internal control in the following cycling conditions: denaturation at 95°C for 30sec, followed by 45 cycles at 95°C for 5sec and 60°C for 34sec. The fold change in gene expression relative to the control was calculated by 2^−∆∆Ct^.

Statistical analysis was performed by unpaired t-testing. As indicated, p < 0.05 was considered significant. Error bars represent means ± SD. The primer sets used in real-time PCR are listed in Table 1.

**Table 1:** 

**Genes**	**Forward Primer 5’-3’**	**Reverse Primer 5’-3’**
**GAPDH**	CGGCAAGTTCAACGGCACAG	CGCCAGTAGACTCCACGACAT
Ca_V_1.2/DCT*	ATCCAAGTTCAGCCGCTACT	GTTGTAGTGTTCGGAGGCAA
**Map 2**	TGTGACTTCCAAATGTGGCT	CAACTTTAGCTTGGGCCTTC
**β-III tubulin**	ACAATGAGGCCTCCTCTCAC	AGGCCTGAATAGGTGTCCAA

* : The primer was designed at the end of C-terminus

### Protein extraction and Western blots

rDPSCs were lysed in RIPA buffer (50 mM Tris–Cl, pH 7.4, 150 mM NaCl, 1% NP40, 0.25% Na-deoxycholate, 1 mM PMSF) with protease inhibitors (in mmol/l: Leupeptin 0.1 and phenylmethylsulfony fluoride 0.3) and kept for 30 min on ice with vortexing every 5 min. The supernatant was collected following centrifugation at 14,000g for 15 min at 4°C. The protein concentration was determined in triplicate by a Bio-Rad DC protein assay Kit. The same amount of proteins (50μg) was loaded for each lane of standard 4–12% SDS–polyacrylamide gels. After electrophoresis, proteins were transferred to a nitrocellulose membrane, and then the membranes were blocked in PBS containing 0.1% Tween and 5% skimmed milk. Membranes were then incubated overnight at 4 °C in 1:200 primary anti-α1C Ca^2+^ channel (Abcam, USA) and DCT antibody (GL Biochem). The immunoblots were developed with horseradish peroxidase-labeled goat anti-rabbit IgG (Chemicon) in PBS-Tween as a secondary antibody (1:2000) for 1 h, followed by detection by ECL (Amersham). 

### Immunofluorescence

Chamber slides were fixed with 4% PFA for 30 minutes at room temperature and then washed with PBS and 0.1% Tween 20 (Sigma-Aldrich) (PBS-T). Cultures were blocked (10% horse serum in PBS-T) for 30 minutes at room temperature and then incubated with primary antibody (1:100 DCT; 1:100 Map 2, Epitomics; 1:200β-III tubulin, Epitomics) in blocking solution overnight at 4°C. Goat, and rabbit controls were treated under the same conditions. After washing, the secondary antibodies (1:200 goat anti-rabbit) were added in blocking solution for 2 hours at room temperature in the dark. Finally, the slides were washed and cover slipped with Prolong gold antifade with 4,6-diamidino-2- phenylindole dihydrochloride (DAPI, P36931; Invitrogen).

## Results

### DCT was detected in rDPSCs

**Figure 1:**
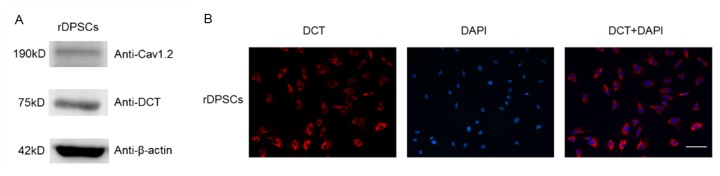
Detection of DCT in rDPSCs. (A). rDPSCs expressing Ca_V_1.2 were probed with anti- Ca_V_1.2 (upper gel) or anti-DCT (middle gel) using western blotting. Anti-DCT recognizes a 75kD short cleavage product that corresponds to the C-terminal fragment of Ca_V_1.2. (B) Immunocytochemistry of rDPSCs grown 6 days in vitro. Anti- DCT staining is shown in red and nuclei are shown in blue. The anti-DCT stained the cell bodies of rDPSCs but no staining was observed in the nuclei. (Scale bars 100μm).

It was reported that DCT translocate to the nucleus of neurons from the brain and regulate directly transcriptions of a variety of genes. To investigate the possible roles of DCT in neural differentiation of rDPSCs, we first developed an antibody to a 14-amino acid peptide in the C terminus of Ca_V_1.2 (aa 2106–2120) and used it to probe rDPSCs expressing Ca_V_1.2. The C-terminal antibody (anti-DCT) recognized a 75kD short cleavage product that corresponds to the C-terminal fragment. Our result confirmed that a closely related C terminal fragment of Ca_V_1.2 in neurons was present in rDPSCs (Figure 1A). 

### Location of endogenous DCT in dental pulp stem cells

Previous studies in cardiac myocytes and neurons have indicated that the DCT fragment translocates to the nucleus and acts as a nuclear transcription factor [20,24]. The intracellular distribution of the DCT fragment in non-excitable cells is unclear. To further investigate the cellular distribution of endogenous DCT in rDPSCs, rDPSCs were stained with anti-DCT antibodies and DAPI, a nuclear label. The anti-DCT stained the cell body of rDPSCs but surprisingly no DCT staining was observed in the nucleus of rDPSCs (Figure 1B).

### Lentivirus transfected rDPSCs

**Figure 2:**
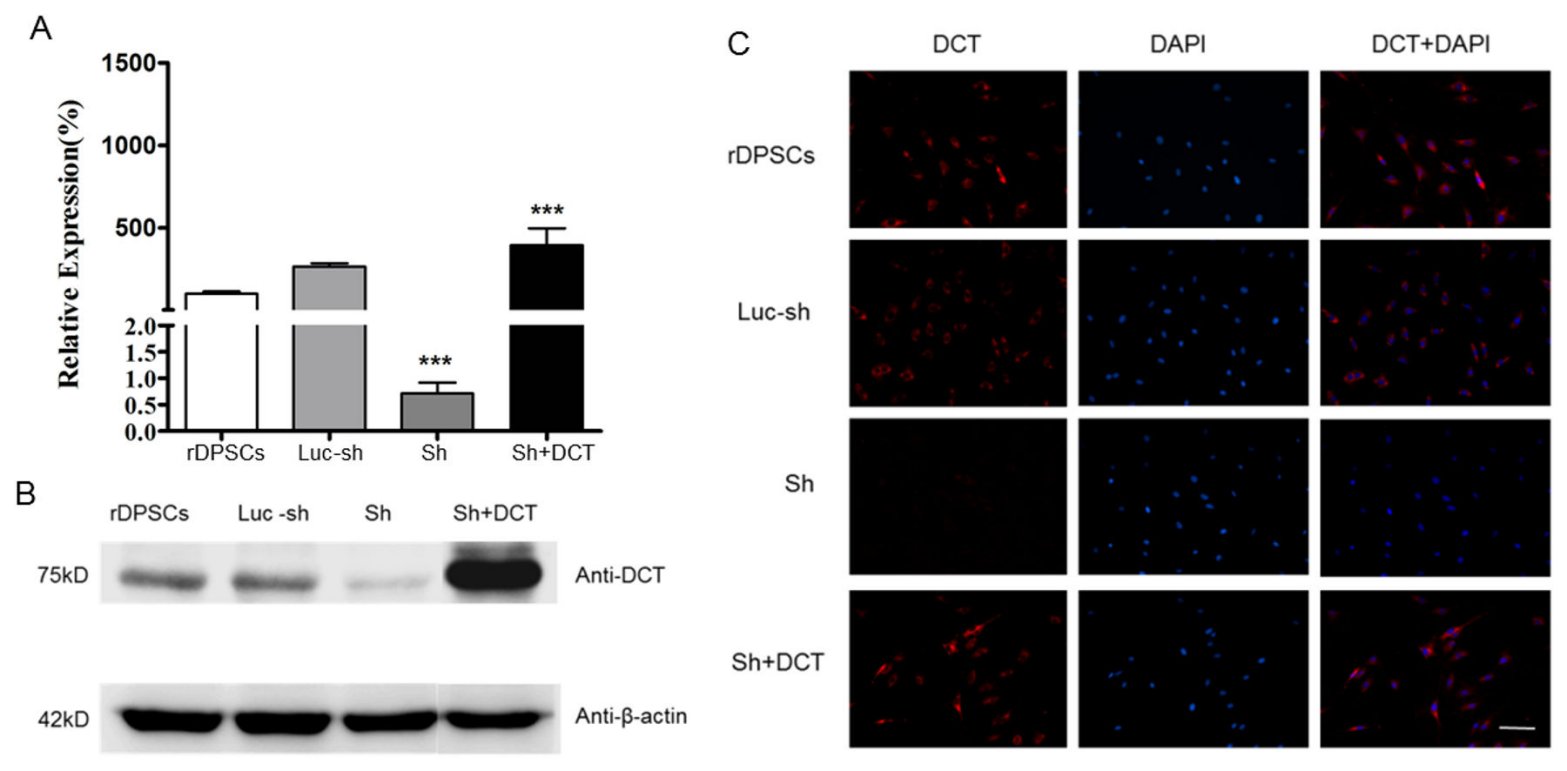
The silencing effect of siRNA on Ca_V_1.2 channels and the rescue effect of DCT in Ca_V_1.2 knockdown rDPSCs. 5 days after transfection, mRNA and protein expression of DCT were analyzed using real-time PCR and western blotting, respectively. The primer was designed at the end of C-terminus of Ca_V_1.2 so that it can detect both the Ca_V_1.2 mRNA and recombinant DCT mRNA. Sh cells: Ca_V_1.2 knockdown cells. Luc-sh cells: luc-shRNA lentiviral control cells. ShSh+DCT cells: DCT-rescued ShSh cells generated by transfecting Ca_V_1.2 knockdown cells with recombinant DCT lentiviral vector. (A): mRNA levels of DCT in Ca_V_1.2 knockdown rDPSCs and DCT-rescued ShSh cells. (B): expression of DCT in Ca_V_1.2 knockdown rDPSCs and DCT- rescued ShSh cells. (C): Immunofluorescence of rDPSCs grown 5 days in vitro. Anti- DCT staining is shown in red and nuclei are shown in blue (Scale bars 100μm).

To investigate the functional significant of DCT in neural differentiation of rDPSCs, a shRNA and lentiviral vectors were used to generate stable Ca_V_1.2 knockdown cells. Recombinant DCT lentiviral vectors were used to transfect Ca_V_1.2 knockdown cells in order to generate DCT-rescued Ca_V_1.2 knockdown cells. We used q-PCR and western blot respectively to detect the transcription and expression of Ca_V_1.2 and recombinant DCT gene. The results showed that Ca_V_1.2 knockdown significantly reduced the mRNA level and protein expression of DCT, but the expression of DCT fragment was significantly increased in rescued Ca_V_1.2 knockdown cells. Figure 2A and Figure 2B represent the mRNA and protein levels of recombinant DCT at day 5 after transfection, respectively. 

### Role of DCT in Neural Induction of rDPSCs in Vitro

**Figure 3:**
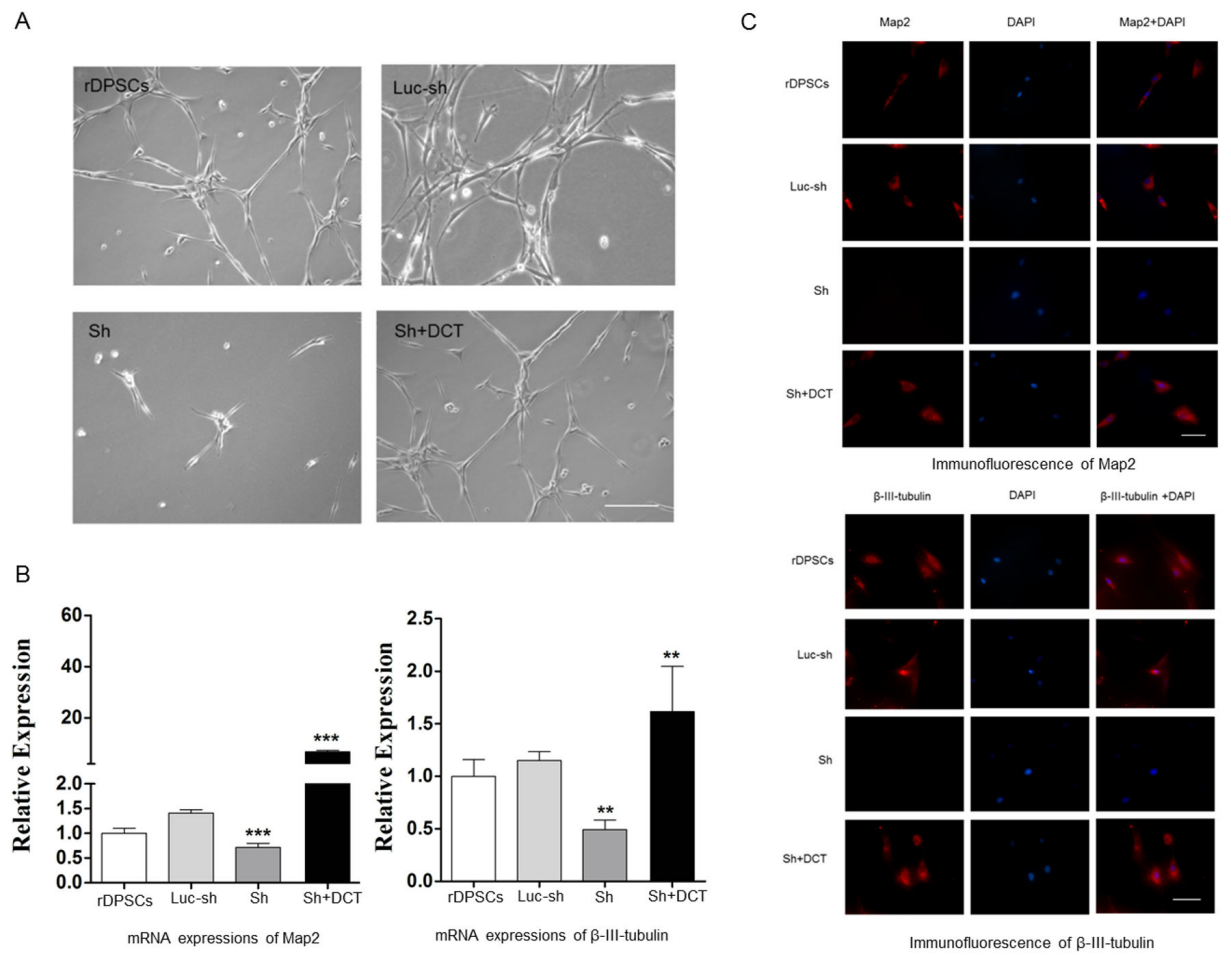
Functional role of DCT in neural differentiation of rDPSCs in vitro. (A): Representative high-magnification images of rDPSCs 14 days after transfection with a vector and shRNA. Neural differentiation was lost in Ca_V_1.2 knock-down rDPSCs but restored to a similar level as the control rDPSCs after DCT expression was rescued (Scale bars 100μm). (B) qRT-PCR and (C) immunofluorescence showed an increase in transcripts and protein expressions of neuronal markers Map2 and β-III-tubulin in rDPSCs cultured in the neuronal inductive conditions. Expressions of neuronal markers were lost in Ca_V_1.2 knock-down rDPSCs but restored to a similar level as the control rDPSCs after DCT expression was rescued (Scale bars 100μm).

In the present study, we used Neurobasal A Media to induce neural differentiation of rDPSCs as previously described [3]. Following 2 weeks of induction, morphological changes of the differentiated cells were observed by phase/contrast microscopy. rDPSCs exposed to neuronal inductive media acquired a bipolar and stellate morphology(Figure 3A), and the control culture predominantly consisted of spindle-shaped cells, indicating rDPSCs cultured in neuronal inductive media had acquired a phenotype resembling mature neurons.

To investigate the functional significance of DCT in rDPSCs, we used shRNA and lentiviral vectors to generate stable Ca_V_1.2 knockdown cells. In Ca_V_1.2 knock-down rDPSCs, neural differentiation was lost (Figure 3A). To determine if the inhibitory effects of Ca_V_1.2 shRNAs on the neural differentiation of rDPSCs are due to reduction of DCT, we constructed a version of DCT that is insensitive to the rat Ca_V_1.2 shRNA and expressed it in Ca_V_1.2 knockdown cells. Expression of DCT rescued the effect of knocking down the endogenous Ca_V_1.2 on the neural differentiation of rDPSCs. 

We further confirmed the effect of DCT on neural differentiation of rDPSCs by assessing the expression of neuron markers. After 14 days of culture in inductive medium, there was a significant increase (*p* < .005 and *p* <.004, respectively, Student *t* test) in the transcripts of Map2 and β-III-tubulin genes in rDPSCs cultured in neural inductive media, compared to the cells maintained in control media (Figure 3B). And at the same time positivity was noted for neuronal markers Map2 and β-III-tubulin (Figure 3C). The transcripts and expressions of neural markers were greatly reduced in Ca_V_1.2 knock-down rDPSCs grown in neural inductive media but restored to similar levels as the control rDPSCs after rescued with recombinant DCT. Changes in transcripts and expressions of neural markers further confirmed the effect of DCT on neural differentiation of rDPSCs. This suggests that endogenous Ca_V_1.2 modulates neural differentiation of rDPSCs and that this differentiation regulation depends on the production of DCT fragment from the C terminus of Ca_V_1.2.

## Discussion

Ca^2+^ entry across the plasma membrane is a main pathway for Ca^2+^ signal. LTCs contribute only a minority of the overall Ca^2+^ entry but exert a dominant role in controlling gene expression. In excitable cells, LTCs is known to play an important role in Ca^2+^ entry across the plasma membrane. However, it’s not clear yet which functions LTCs perform in non-excitable cells, especially in the undifferentiated stem cells. Critical role of LTCs in neural stem/ progenitor cell differentiation has been attributed to LTCs-mediated Ca^2+^ influx. It was reported that Ca^2+^ entry through LTCs mediated hypoxia-promoted proliferation of neural progenitor cells [25]. Calcium influx via Ca_V_1 channels supports sustained phosphorylation of cAMP response element-binding protein (CREB) and CREB-dependent gene expression in neurons [13-16]. In the present study, knock-down of Ca_V_1.2 channels significantly decreased neural differentiation of rDPSCs, and restoring expression of DCT in Ca_V_1.2 knock-down rDPSCs rescued neural differentiation of these dental stem cells. This result implied the marked inhibition of neural differentiation in Ca_V_1.2 knock-down DPSCs is not a result of decreased Ca^2+^ influx through Ca_V_1.2 channel, but because of the reduction of DCT from Ca_V_1.2. 

Ca^2+^ oscillations are mainly determined by two sources: Ca^2+^ entry across the plasma membrane and Ca^2+^ release from intracellular stores. Although the physiological functions of Ca^2+^ oscillations in stem cells are still unknown, some studies about Ca^2+^ signaling pathway in human mesenchymal stem cells showed that in human mesenchymal stem cells, unlike in excitable cells, it was not the Ca^2+^ entry through plasma membrane but the Ca^2+^ release from intercellular store that plays an important role in [Ca^2+^]i oscillations [26,27]. Zahanich et al. also found that L-type Ca^2+^ channel blocker did not influence alkaline phosphatase activity, [Ca^2+^]i, and phosphate accumulation in human mesenchymal stem cells during osteogenic differentiation, suggesting that osteogenic differentiation of human mesenchymal stem cells did not require L-type Ca^2+^ channel function[28]. The results of our study are consistent with those of previous studies on [Ca^2+^]i oscillations in human mesenchymal stem cells. In the present study, other types of Ca^2+^ channels, and/or internal Ca^2+^ stores may significantly contribute to [Ca^2+^ ]i elevation in those Ca_V_1.2 knock-down DPSCs. But this speculation needs more examination in a future study. 

Proteolytical cleavage of LTCs at their C terminus has been reported in excitable cells, such as cardiac and skeletal muscle cells and neurons [20,24,29,30]. In heart and skeletal muscle, the C-termini of Ca_V_1.2 and Ca_V_1.1 are proteolytically cleaved and produce a ~45kDa fragment, which plays an important role in regulating channel properties and trafficking the channel to the plasma membrane. Dolmetsch’s laboratory reported that the entire C-terminal of Ca_V_1.2, appearing as a ~75kDa band in western blots, translocated to the nucleus of neurons and regulated transcriptions of a variety of genes [20]. In the present study a ~75kDa fragment was detected in rDPSCs, suggesting the full length C-terminal fragment of Ca_V_1.2 is proteolytically cleaved in rDPSCs, in the same manner as it is in neurons. According to our knowledge, this is the first study reporting that cleaved C-termini of LTC channels are present in non-excitable cells. It’s still not known where the cleavage site is and how this process is regulated. And it is also possible that alternative splicing of the Ca_V_1.2 gene may independently generate DCT [31]. These questions are important areas for future studies.

We also found in the present study that the level of DCT was low in rDPSCs and there was no DCT detected in nuclei of these cells before neural induction. This result is contrary to that of Dolmetsch’s studies in neurons [20], which showed the cleaved Ca_V_1.2 C-terminal translocated to the nucleus and acted as a transcriptional factor. But Ca_V_1.2 knock-down rDPSCs have a nearly complete loss of neural differentiation and re-expression of DCT in Ca_V_1.2 knock-down rDPSCs can rescue the neural differentiation of rDPSCs, suggesting a general requirement of DCT in the neural differentiation of rDPSCs. It’s possible that in the present study DCT from Ca_V_1.2 regulate neural differentiation of rDPSCs through indirect way other than acting as a transcription factor itself, for example activating such transcription factors as CREB and nuclear factor of activated T-cell (NFAT) and indirectly regulate expression of genes related with cell differentiation. It will also be interesting in future studies to determine the cellular distribution of DCT in DPSCs and the molecular mechanisms of its action in neural differentiation of dental stem cells. 

Our study provides strong evidence that distal C-terminal fragment of Ca_V_1.2 is present in rDPSCs and it acts as an important regulating factor in the neural differentiation of rDPSCs. Further studies concerning the possible mechanisms how DCT is produced and how it regulates cell differentiation would be of utmost importance to realize the use of these stem cells in nerve regeneration.
